# Transcatheter Arterial Coil Embolization of Ruptured Common Hepatic Artery Aneurysm in a Patient with Behçet's Disease

**DOI:** 10.1155/2015/790175

**Published:** 2015-03-04

**Authors:** Akihiro Hotta, Ryohei Kuwatsuru, Kouichi Asahi, Shingo Okada, Daisuke Tsuge, Akihiko Shiraishi, Yoshinari Takasaki

**Affiliations:** ^1^Departments of Radiology, Juntendo University Faculty of Medicine, 2-1-1 Hongo, Bunkyo-ku, Tokyo 113-8421, Japan; ^2^Departments of Internal Medicine and Rheumatology, Juntendo University Faculty of Medicine, 2-1-1 Hongo, Bunkyo-ku, Tokyo 113-8421, Japan

## Abstract

Hepatic artery aneurysm is a rare and potentially life-threatening entity. We report a case of ruptured common hepatic artery aneurysm in a patient with Behçet's disease. The ruptured aneurysm was treated successfully with transcatheter arterial coil embolization. Transcatheter arterial embolization is the preferred treatment modality in patients at high risk of surgical intervention.

## 1. Introduction

Behçet's disease is a multisystem disorder characterized by recurrent ulcers of the mouth and genitalia and relapsing iritis. It was first described by the Turkish dermatologist Hulusi Behçet in 1937 and most often affects men between 20 and 40 years of age. The disease is most prevalent in the Mediterranean region, Middle East, and far east. The cause of Behçet's disease is unclear. Vascular involvement appears in 5% to 30% of patients [1] and manifests as venous occlusion, aneurysm, arterial occlusion, and varices [2].

We describe a case in which we successfully used transcatheter arterial coil embolization to treat a ruptured common hepatic artery (CHA) aneurysm in a patient with Behçet's disease.

## 2. Case Report

A 34-year-old man presented to our hospital with acute right abdominal pain. He had been diagnosed with Behçet's disease 10 years before. The patient had undergone aortic replacement with celiac and superior mesenteric artery restoration for aortic dissection 18 months previously. He also had undergone ligation for aneurysms of the gastroduodenal artery and both renal arteries. He had not taken steroid before and after surgery. He had been receiving hemodialysis for the past 18 months. He also had hypertension.

**Figure 1:**
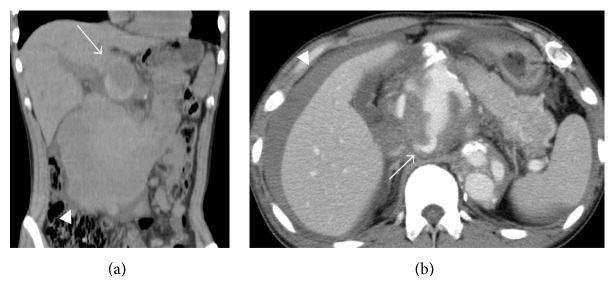
(a) Nonenhanced CT shows an aneurysm (arrow) of the common hepatic artery, with a hematoma (arrowhead) extending inferiorly. (b) Contrast-enhanced CT two and a half hours later reveals extravasation (arrow) from the aneurysm and hematoma extending to the lateral aspect of the right lobe of the liver (arrowhead).

Nonenhanced CT showed a 6 cm aneurysm of the CHA, with a hematoma extending inferiorly (Figure 1(a)). Contrast-enhanced CT two and a half hours later revealed extravasation from the aneurysm, with the hematoma now extending to the lateral aspect of the right lobe of the liver (Figure 1(b)). The right and left hepatic arteries branched individually from the aneurysm. The portal vein was patent.

**Figure 2:**
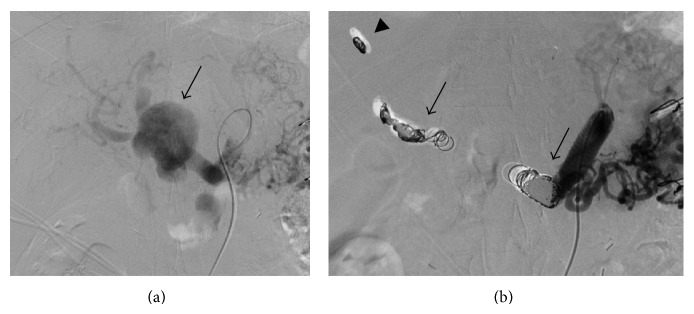
(a) Celiac angiography demonstrates a saccular aneurysm (arrow) 2 cm distal to the origin of the common hepatic artery (CHA). (b) Celiac angiography after embolization of the right hepatic artery and CHA (arrow) shows the occluded aneurysm. Incidentally migrated coils (arrowhead) are seen in the distal part of the RHA.

Angiography and embolization of the aneurysm were then planned. A 4-Fr Cobra catheter (Selecon PA, Terumo Clinical Supply, Gifu, Japan) was placed at the celiac artery via a femoral artery approach. Celiac angiography showed a saccular aneurysm 2 cm distal to the origin of the CHA (Figure 2(a)). A 2-Fr microcatheter (Masters Parkway, Asahi Intecc, Aichi, Japan) was placed at the right hepatic artery (RHA). Twenty-one microcoils (Tornado, Cook Medical, Bloomington, IN) were deployed in the RHA. The proximal side of the coils was deployed in the aneurysm; several coils migrated to the distal part of the RHA during embolization. We tried to cannulate the left hepatic artery (LHA) but it was unsuccessful. The CHA then was embolized by using 17 Guglielmi detachable coils (Stryker, Kalamazoo, MI). Celiac angiography after embolization showed the occluded aneurysm (Figure 2(b)).

The patient developed a fever after embolization. There was no evidence of liver necrosis nor severe liver dysfunction without elevation of liver transaminases. His fever abated after intravenous antibiotic administration. Abdominal CT 2 weeks after the treatment showed that the hematoma had decreased greatly, and there was no evidence of active bleeding. Abdominal CT 1 year after the treatment documented the disappearance of the hematoma and gave no evidence of residual or recurrent aneurysms. Clinical and CT follow-up at three and half years after the treatment showed no new abnormalities and well-healed treatment sites.

## 3. Discussion

Behçet's disease is a multisystemic disorder that affects the mucocutaneous, ocular, articular, neurologic, cardiovascular, gastrointestinal, and respiratory systems. Vascular involvement occurs in 5% to 30% of patients with Behçet's disease [1] and manifests as venous occlusion, aneurysm, arterial occlusion, and varices [2]. In vascular involvement, arterial lesions are less frequent than venous lesions; arterial lesions account for only 12% of vascular involvement in Behçet's disease [3]. However, the leading cause of death in patients with Behçet's disease is the rupture of large-artery aneurysms [2]. Aneurysms are present in 65% of patients with arterial lesions and arterial occlusions in 35% [2]. The most common site of aneurysm is the abdominal aorta, followed by the femoral and pulmonary arteries [2]. Hepatic artery aneurysms have been reported infrequently [3–6].

In general, hepatic artery aneurysm (HAA) is the second most frequent type of visceral aneurysm after splenic artery aneurysm [7, 8] and is two times more likely to occur in men than women. Most (80%) HAA are extrahepatic in location and affect the CHA in 63% of cases, the RHA in 28%, the LHA in 5%, and the RHA and LHA concurrently in 4% of cases [7]. Most HAA are solitary lesions [8]. Atherosclerosis accounts for an estimated 30% of cases [7]; vasculitis, fibromuscular dysplasia, trauma, and iatrogenic injury are other well-known causes [7, 8]. Approximately 14% to 80% of all HAAs rupture [9]. Criteria for treatment include aneurysms that are ruptured, symptomatic, multiple, or nonatherosclerotic. Atherosclerotic aneurysms larger than 2 cm are also recommended intervention [9]. During the last decade, the technique most frequently used (37% of cases) for treatment of HAA has been transcatheter embolization. To treat these lesions surgically, ligation was performed in 36% of cases, aneurysmectomy in 27%, and bypass/revascularization in 15% [7].

The basic pathogenesis underlying the vascular complications of Behçet's disease is vasculitis of the vasa vasorum in medium and large vessels [4, 10]. The large arterial lesions involve inflammation of the media and adventitia. In affected arteries, active arteritis occurs initially, followed by medial destruction and fibrosis. Saccular aneurysms likely arise from severe medial destruction due to intense inflammation [10]. Because of the fragility of the walls of the involved vessels and aneurysms in Behçet's disease, surgical repair of these aneurysms is often unsuccessful and may result in new aneurysm formation and graft occlusion [3, 11]. In our patient, we considered that the CHA aneurysm was a pseudoaneurysm that formed near the ligation site of the gastroduodenal artery aneurysm.

Transcatheter embolization is a reasonable alternative to traditional surgical approaches to prevent complications or recurrences and is considered to be safe and effective for arterial aneurysms in the context of Behçet's disease. A single review and several reports support it [3–6, 12]. The success rate of transcatheter embolization ranges from 70% to 100% [7]. Complications of embolization include rupture, liver necrosis, abscess formation, infection, sepsis, ascites, jaundice, gallbladder necrosis, and the formation of a pseudoaneurysm or hematoma at the catheter site [8].

In the single report of aneurysm of the CHA with a fistulous communication with the superior mesenteric vein in a patient with Behçet's disease [5], transcatheter embolization proceeded using N-butyl cyanoacrylate and lipiodol. We believe that our current report is the first to describe the use of transcatheter arterial coil embolization to treat a ruptured CHA aneurysm in a patient with Behçet's disease. In this patient, we used microcoils for the isolation technique. We used pushable coils for the embolization of the RHA. In addition, we selected Guglielmi detachable coils for embolization of the CHA because of its rapid blood flow. Stenting might be considered as another treatment. However, it was considered difficult, because calibers of proximal and distal part of the CHA were considerably different.

In summary, we successfully performed transcatheter arterial coil embolization to treat a ruptured CHA aneurysm in a patient with Behçet's disease. Transcatheter arterial embolization is the preferred treatment modality for patients at high risk of complications during and after surgery.
